# Autonomic and Renal Alterations in the Offspring of Sleep-Restricted Mothers During Late Pregnancy

**DOI:** 10.6061/clinics/2016(09)07

**Published:** 2016-09

**Authors:** Joyce R S Raimundo, Cassia T Bergamaschi, Ruy R Campos, Beatriz D Palma, Sergio Tufik, Guiomar N Gomes

**Affiliations:** I Escola Paulista de Medicina – UNIFESP, Departamento de Fisiologia, São Paulo/SP, Brazil; II Escola Paulista de Medicina – UNIFESP, Departamento de Psicobiologia, São Paulo/SP, Brazil; III Centro Universitário São Camilo, São Paulo/SP, Brazil

**Keywords:** Prenatal Exposure Delayed Effects, Hypertension, Kidney Disease, Sleep Restriction

## Abstract

**OBJECTIVES:**

Considering that changes in the maternal environment may result in changes in progeny, the aim of this study was to investigate the influence of sleep restriction during the last week of pregnancy on renal function and autonomic responses in male descendants at an adult age.

**METHODS:**

After confirmation of pregnancy, female Wistar rats were randomly assigned to either a control or a sleep restriction group. The sleep-restricted rats were subjected to sleep restriction using the multiple platforms method for over 20 hours per day between the 14^th^ and 20^th^ day of pregnancy. After delivery, the litters were limited to 6 offspring that were designated as offspring from control and offspring from sleep-restricted mothers. Indirect measurements of systolic blood pressure (BPi), renal plasma flow, glomerular filtration rate, glomerular area and number of glomeruli per field were evaluated at three months of age. Direct measurements of cardiovascular function (heart rate and mean arterial pressure), cardiac sympathetic tone, cardiac parasympathetic tone, and baroreflex sensitivity were evaluated at four months of age.

**RESULTS:**

The sleep-restricted offspring presented increases in BPi, glomerular filtration rate and glomerular area compared with the control offspring. The sleep-restricted offspring also showed higher basal heart rate, increased mean arterial pressure, increased sympathetic cardiac tone, decreased parasympathetic cardiac tone and reduced baroreflex sensitivity.

**CONCLUSIONS:**

Our data suggest that reductions in sleep during the last week of pregnancy lead to alterations in cardiovascular autonomic regulation and renal morpho-functional changes in offspring, triggering increases in blood pressure.

## INTRODUCTION

During intrauterine development, fetal organs and tissues go through developmental periods designated as critical periods, in which cells undergo intense division 1. Alterations during these critical periods may cause fetal adaptations or “fetal programming” that result in lifelong consequences related to metabolic and cardiovascular changes 2-4.

Sleep restriction (SR) seems to affect essential mechanisms required for the maintenance of homeostasis, resulting in disorders such as hypertension 5-7, glucose intolerance and increased production of various hormones such as corticosterone, growth hormone (GH) and adrenocorticotropic hormone (ACTH), among others 8-11. The mechanisms underlying such alterations are not yet clear; however, increases in sympathetic nervous system activity and hypothalamic-hypophysis-adrenal axis activity appear to be related to the changes observed after SR 11,12. Studies performed in humans have shown that sleep deprivation of about 24–26 h is enough to alter arterial baroreflex function 7 and cardiac sympathetic modulation 13, increasing blood pressure values 14. These data support the notion that autonomic misbalance is related to the changes caused by SR.

SR is a global phenomenon related to modern lifestyle that affects both men and women 15. During pregnancy, anatomical and physiological alterations are related to the onset of sleep disorders 16,17. Furthermore, SR associated with changes resulting from pregnancy may be harmful to both maternal and fetal health 17,18. Despite this, few studies have assessed the impact of SR during pregnancy on offspring. Alvarenga et al. 19 observed that the progeny of rats subjected to SR during pregnancy presented hormonal changes and prejudicial sexual responses in adulthood. Radhakrishnan et al. 20 showed that SR in late pregnancy caused anxiety-related behavioral alterations in young offspring. Considering that renal development may be affected by insults during pregnancy 21, we studied the effects of SR both in late pregnancy and throughout pregnancy on renal morphology and function 21,22. The consequences of SR during the last week of pregnancy, a period critical for kidney development, were studied by Thomal et al. SR during this stage caused reductions in nephron number and augmented blood pressure in offspring 21. Lima et al. showed that SR throughout pregnancy did not produce clear renal morphological changes but did alter the sensitivity of the cardiac baroreflex response, suggesting that autonomic regulation of blood pressure was affected 22. The present study aimed to assess what effects SR at the end of pregnancy has on kidney development and autonomic regulation of blood pressure.

## MATERIALS AND METHODS

This study was evaluated and approved by the Ethical Research Committee of the Universidade Federal de São Paulo - UNIFESP (CEUA: 7647020614) and adhered to international guidelines for the care of research animals.

### Experimental Groups

Female (weighing 200-250 g) and male (weighing 300-350 g) three-month-old Wistar rats were used in this study. The animals (12 female and 6 male) could freely access food and water throughout the experimental protocol and were housed in a room with temperature and humidity control (21±2°C, 60%) and a light/dark cycle of 12:12 h, with lights on at 07:00.

### Pregnancy Confirmation

Two females spent the night with one male and vaginal discharge was collected the next morning. The presence of sperm was regarded as a positive result and considered day zero of pregnancy. The females were then randomly assigned to either the control mothers group or the SR mothers group.

### Sleep Restriction Protocol

The SR technique was based on the muscle atony that accompanies paradoxical sleep 23. Briefly, 10 narrow, circular platforms (6.5 cm in diameter) were placed inside a tiled tank (123×44×44 cm) filled with water to within 1 cm below the upper border of the platforms. For the SR mothers, 2 to 6 rats were placed on the platforms in an arrangement that allowed them to move inside the tank and jump from one platform to the other. Two days before the beginning of the study, the animals were adapted to the water tank for a period of 1 h to avoid unnecessary falls into the water. The SR mothers were placed in the tank between the 14^th^ and 20^th^ day of pregnancy for 20 hours a day (from 2 pm until the next day at 10 am). After this period, they were returned to their home cages and could sleep freely. At 10 am on the 20^th^ day of pregnancy, the SR mothers were placed back into their home cages for maintenance until spontaneous delivery and weaning of the offspring. The control group was housed in the same area in which the sleep deprivation took place.

### Birth and Weaning

After birth, the animals were weighed, and six litters, with a proportion of four males to two females, stayed with their mothers for 28 days. From the 28^th^ of life, the offspring were separated from their mothers, and the male offspring were placed in collective cages containing four animals per cage. The females were not used in the present study. The offspring were distributed into two groups: a control mothers offspring (CO) group and a SR mothers offspring (SRO) group.

The four male offspring from each litter were used in different experimental procedures (i.e., analyses of renal function, cardiac baroreflex analysis, and double pharmacological blockage) to avoid having siblings undergo the same evaluations. Initial renal function measurements were performed when the rats were three months old. Cardiovascular parameters were obtained sequentially. Because the renal experimental and analysis procedures used in this study are time-consuming, cardiovascular evaluations were performed when the rats were four months old.

### Indirect Determination of Systolic Blood Pressure

At two months of age, the offspring began adaptation to a tail plethysmography apparatus. Effective determination of indirect systolic blood pressure (BPi) was obtained at 3 three months of age. The animals were placed in acrylic cylinders with appropriate dimensions for the size of the animal while the tail remained exposed. The sphygmomanometer had a sensor connected to a register system (Monitor Ratpalp.b) and was adjusted to the proximal tail portion of the rat. Three measurements were performed in sequence; the mean of these measurements was considered the BPi.

### Renal Function Studies

Clearance evaluations were performed when the animals were three months old. First, anesthesia was induced with sodium thiopental (90 mg/kg). Next, the trachea was catheterized to maintain adequate ventilation and the carotid artery and jugular vein were catheterized for infusions and for blood sampling, respectively. The bladder was catheterized for urine collection. Following this, the animals received an infusion solution (0.9% sodium chloride plus 3% mannitol) delivered at a constant rate. The animals received a primer solution (1 ml of saline containing inulin, 300 mg/kg and sodium para-aminohippurate (PAH) 6.66 mg/kg) and then a continuous infusion (saline containing inulin, 5 mg/min/kg and PAH, 1.33 mg/min/kg) at 0.1 ml/min. Inulin and PAH concentrations were measured by colorimetry in plasma and urine samples to estimate glomerular filtration rate (GFR) and renal plasma flow (RPF). The urinary excretion of titratable acid (TA) was measured by microtitration. The excreted amount of ammonium (EANH4) was evaluated by colorimetry as described previously 24. Sodium (Na^+^) and potassium (K^+^) concentrations were determined with a flame photometer, and after obtaining the concentrations of these ions in plasma and urine, the filtered load (FL), excreted load (EL) and fractional excretion (FE%) were calculated.

### Renal Morphometric Analysis

After the clearance evaluation, the rats were euthanized and their kidneys were removed and immersed in Bouin’s solution for fixation. The kidneys were cut lengthwise, embedded in wax, and sliced into 5-μm-thick sections. The sections were stained with hematoxylin and eosin and analyzed using a trinocular microscope. Images (200x magnification) were acquired using a Nikon microscope (Nikon H550 L) connected to a microcomputer and a Nikon DS-Ri1 video camera. Twenty-five cortical fields (with an area of 277,000 μm^2^) were analyzed; the glomeruli were counted and measured to determine their area and the results are expressed as the glomeruli per field and μm^2^, respectively 22,24.

### Cardiovascular Parameters

Cardiovascular evaluation was performed when the animals were four months old. The animals were anesthetized with xylazine (4 mg/kg) and ketamine (100 mg/kg) and their femoral veins and arteries were catheterized for drug administration and to obtain the following cardiovascular parameters: mean arterial pressure (MAP), systolic blood pressure (SBP), diastolic blood pressure (DBP) and heart rate (HR) 25. The ends of the catheters were externalized in the neck region of the animal, with the aid of a guide catheter. The functional experiments were performed 24 hours after surgical recovery. MAP, SAP, DAP and HR were recorded online using an analog-digital converter board (PowerLab AD Instruments).

### Cardiac Baroreflex Analysis

To analyze the cardiac baroreceptor reflex in awake animals, increasing doses of phenylephrine (1-3 μg/kg, iv) (Sigma-Aldrich) were acutely administered to increase blood pressure and cause reflex bradycardia 25. To reduce blood pressure and induce reflex tachycardia, increasing doses of sodium nitroprusside (2, 5 and 7 μg/kg, iv) were administered. The cardiac baroreflex was evaluated by the mean index relating changes in HR to changes in MAP and expressed as beats per mmHg as previously described 25.

### Double Pharmacological Blockage Analysis

Autonomic regulation of the heart was analyzed through recording changes in HR after selective pharmacological blockade of the parasympathetic and sympathetic nervous system using anti-cholinergic and beta-blocker drugs, respectively 26. The bradycardic response obtained after β-adrenergic receptor blockade with atenolol (1 mg/kg, i.v.; Sigma-Aldrich Co, St Louis, MO, USA) was used to estimate sympathetic tone (Atenolol Δ). The tachycardia response after muscarinic cholinergic receptor blockade with methyl atropine (3 mg/kg, i.v.; Sigma-Aldrich Co, St Louis, MO, USA) was used to estimate vagal tone (Atropine Δ). At the end of the experiment, hexamethonium bromide, a ganglion blocker that also inhibits the effects of noradrenalin on vessels, was slowly administered (1 mg/kg, iv; Sigma-Aldrich Co, St Louis, MO, USA). The sympathetic tone to the vessels was considered the difference between the minimum MAP obtained after hexamethonium blockade and the basal MAP (Hexamethonium Δ).

### Spectral Analysis

For spectral analysis of HR and systolic pressure variability, the beat-by-beat HR and systolic arterial pressure were recorded over a 10-min period in conscious rats. Fast Fourier transformation (FFT) was used to calculate the spectral densities of the frequency components of the HR and systolic pressure 26. The HR and systolic pressure data were converted every 100 ms with a cubic spline interpolation (10 Hz). The interpolated series were divided into half-overlapping sequential sets of 512 data points (51.2 s). The segments were inspected visually and nonstationary data were discarded. A Hanning window was used to attenuate the side effects. The power intensity was computed using a direct FFT algorithm for discrete time series. The total power in the low frequency band (LF: 0.2-0.75 Hz) and high frequency band (HF: 0.75-3 Hz) was calculated. The LF/HF power ratio was calculated and used as an indicator of cardiac sympathovagal balance 27. The basal parameters of systolic arterial pressure and HR were registered 24 h after femoral catheterization and before the analysis of cardiac baroreflex or double pharmacological blockage.

### Statistical Analysis

Data are expressed as the means±SE. Statistical analysis was performed using the program Prism (GraphPad Software). The normality test, the Kolmogorov-Smirnov test and the comparative unpaired Student’s *t*-test were used. For data that did not present a normal distribution, the Mann Whitney comparative test was used. P≤0.05 was considered significant.

## RESULTS

### Body Size Measurements

No significant differences were observed in body weight measured at 1, 2 and 3 months (Table 1). The naso-anal length (NAL) was measured monthly; at three months, the SRO group presented a decrease in NAL in comparison to the CO group.

### Systolic Blood Pressure

At three months, the SRO group had significantly increased BPi values compared to the CO group (CO: 122.2±0.74; SRO: 144.6±0.94* mmHg).

### Renal Function Analysis

GFR was significantly increased in the SRO group compared to the CO group (Table 2). However, RPF and urinary flow did not differ between the groups (V: CO: 0.11±0.02; SRO: 0.10±0.01 ml/min/kg). Sodium, potassium and acid excretion were similar in both groups.

### Morphological Parameters for the Kidney

**Figure 1:**
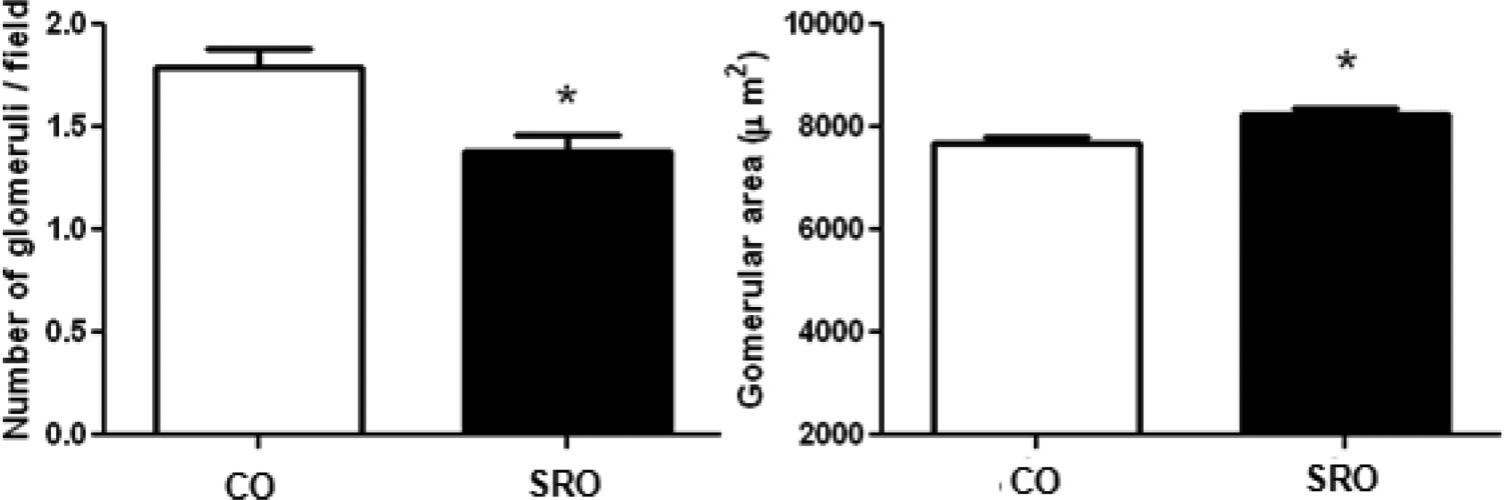
Number of glomeruli/field and glomerular area of the studied groups. CO = control mother offspring; SRO = sleep-restricted mother offspring. *p≤0.05 *vs.* CO group (Students T test).

Under microscopic examination, the SRO rats presented a significant decrease in the glomeruli counted per field compared to the CO rats. Furthermore, the SRO rats also presented a significant increase in the glomerular area (Figure 1).

### Cardiovascular Function

Direct measurements of cardiovascular parameters were performed at four months. The SRO group demonstrated significant increases in SAP, MAP and HR compared to the CO group. However, the DAP in the SRO rats was not significantly different from that in the CO rats (SAP: CO: 124±1.7; SRO: 139±3.02* mmHg; MAP: CO: 103±1.4; SRO: 110±2.7* mmHg; DAP: CO: 91±1.5; SRO: 96±2.9 mmHg; HR: CO: 335±4.5; SRO: 354±7.5* bpm).

### Cardiac Baroreceptor Reflex Sensitivity

**Figure 2:**
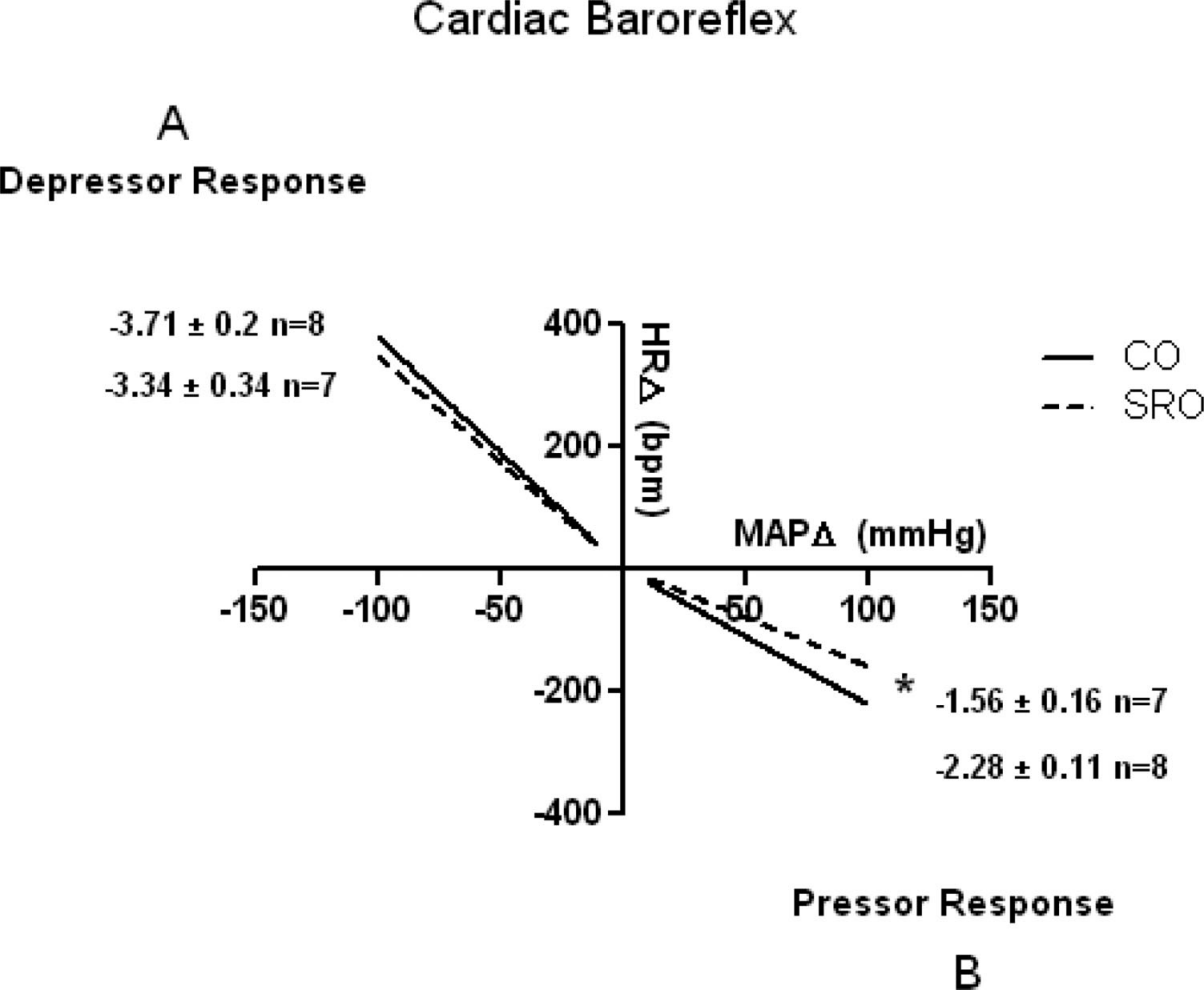
Cardiac baroreflex sensibility in the studied groups. (A) Depressor response induced by sodium nitroprusside. (B) Pressor response induced by phenylephrine. CO = control mother offspring; SRO = sleep-restricted mother offspring. *p≤0.05 *vs.* CO group

The SRO rats demonstrated significant impaired baroreflex sensitivity (bradycardic reflex response) for sudden elevations in MAP. However, the tachycardic reflex response induced by sodium nitroprusside administration showed no difference between the groups (Figure 2).

### Sympathetic and Parasympathetic Tone

The bradycardia observed following the administration of atenolol (Atenolol Δ) was significantly greater in the SRO rats compared to the CO rats. However, the tachycardic response observed after atropine administration (Atropin Δ) was not different between the SRO and CO groups (Table 3).

### Balance of Sympatho-Vagal Tone

Table 4 shows the results of spectral analysis for the pulse interval (PI) and arterial pressure (AP). The LF values for the PI and AP were significantly higher in the SRO group compared to the CO group. The HF value for PI was decreased and the LF/HF ratio was increased in the SRO group, suggesting a sympatho-vagal misbalance with increased sympathetic modulation.

## DISCUSSION

The present study revealed that SR during the last week of pregnancy reduced baroreflex response sensitivity, increased sympathetic tone to the heart and caused sympatho-vagal misbalance in adult male offspring. This study also confirmed that these offspring present elevated blood pressure values and renal morpho-functional changes, as described by Thomal et al. 21.

The participation of the kidneys in the development of hypertension has long been suggested and numerous mechanisms have been proposed to confirm this hypothesis 28,29. In recent decades, the influence of inadequate intrauterine growth over nephron formation has also been associated with the development of hypertension 30-32.

Similarly to nutritional or growth restriction models 33,34, the SRO group exhibited a significantly reduced number of nephrons in comparison to the CO group and also presented glomerular hypertrophy; however, at the ages that the animals were studied, no decreases in GFR or in sodium excretion were observed, suggesting that renal adaptations in the SRO animals may be responsible for dislocation of the pressure-diuresis curve 35. In fact, increased GFR was observed in the SRO rats. Under normal conditions, GFR is maintained within narrow limits even when blood pressure changes due an auto-regulatory mechanism that acts by changing the resistance of glomerular arterioles. However, this mechanism may be weakened during hypertension and increased blood pressure in the glomerular capillaries may be responsible for enhancing the GFR 36. It is possible that these changes occurred in the SRO group; however, further experiments are necessary to corroborate this hypothesis.

To analyze whether changes in the central regulation of blood pressure contributed to the increased blood pressure in the SRO rats, we assessed autonomic cardiac function in this experimental model. Reduced baroreflex sensitivity, increased sympathetic tone to the heart and sympatho-vagal misbalance with an increase in sympathetic modulation were found in the adult SRO animals.

Impairment in the baroreflex response has been shown in diseases such as diabetes and hypertension 37,38. However, the role of baroreceptors in the cardiovascular system in chronic disease is not clear, especially considering that after approximately two days of hypertension the adaptation of baroreceptors is completed 39. However, in parallel to baroreceptor adaptation, there is also a reduction in baroreflex sensitivity, which directly affects blood pressure regulation 40.

Reduced arterial baroreflex sensitivity may induce an increase in efferent sympathetic vasomotor activity because, under physiological conditions, the activity of aortic and carotid receptors results in an inhibitory effect on sympathetic activity 40. Thus, in our experiments, the increase in sympathetic modulation found in the SRO rats may be related to a reduction in arterial baroreflex sensitivity, triggering sympatho-excitation and leading to blood pressure increase. However, the mechanisms underlying the baroreceptor dysfunction found in the SRO group remain unclear and require further investigation. Altered baroreflex sensitivity and sympathetic activity have also been observed in offspring subjected to prenatal exposure to dexamethasone 41,42, confirming the role of fetal exposure to corticosteroids in autonomic alterations in later life.

Interestingly, the progeny of rats subjected to nutritional restriction during pregnancy also exhibit cardiac baroreflex impairment and increased expression of angiotensin II receptor type 1 (AT1) in brain regions responsible for blood pressure regulation 43. These changes are probably related to alterations in efferent autonomic baroreflex pathways. Increased AT1 receptor expression may be a consequence of positive feedback due to increased Ang II in the central nervous system. This is because in rats, the chronic intracerebroventricular infusion of Ang II leads to increased expression of AT1 receptors and AT1 mRNA in the brain 44. Other studies have associated increased hypothalamic expression of AT1 receptors with the use of glucocorticoids (dexamethasone) during pregnancy 45,46. In this experimental model, hypertension and decreased baroreflex sensitivity in offspring were also observed 41,42,47. Furthermore, the use of AT1 receptor inhibitors in late pregnancy attenuated baroreflex impairment and blood pressure increases in offspring 47, suggesting that changes in the expression of RAS components during development can alter central structures involved in blood pressure regulation 47. Therefore, we cannot exclude the possibility that sleep deprivation during pregnancy may alter the expression of Ang II components within the brain, leading to cardiovascular alterations as previously reported 22,48

The alterations observed in the SRO group may be related to corticosterone expression. Several genes are modulated by glucocorticoids and fetal exposure to non-physiological concentrations of these hormones may result in the improper programming of these genes 49. During pregnancy, the enzyme 11-β-hydroxysteroid dehydrogenase (11bHSD) converts corticosterone (in rats) into non-active metabolites 49. However, in late pregnancy, the level of this enzyme is reduced and cortisol (or corticosterone) levels can increase during this period and reach the developing fetus 49.

The modulation of several hormones may be modified by SR or sleep deprivation 8,9. Increased secretion of ghrelin, ACTH, cortisol and GH after sleep deprivation was shown by Schussler et al. 9. During early pregnancy in mice, sleep deprivation caused a significant decrease in progesterone and an increase in corticosterone plasma concentration 50. Sleep deprivation in rats also increases the concentrations of corticosterone and norepinephrine in plasma and the secretion of hypothalamic hormones such as prepro-orexin (PPO) and neuropeptide Y (NPY) 10.

During critical periods of development, fetal exposure to non- physiological concentrations of hormones may promote hypothalamic dysfunction in offspring 51. This perinatal epigenetic programming phenomenon induced by hormonal changes during development was initially proposed by Günter Dörner. In this theory, hormones play a decisive role because they are influenced by the environment and also modulate neuroendocrine regulatory systems, which control all fundamental processes of life 52.

Thus, SR at the end of pregnancy influences the development of offspring through hormonal changes, causing adaptations that result in autonomic and kidney abnormalities that can be observed in the progeny during adulthood. However, additional research is needed to understand the mechanisms underlying the modifications observed in the present study.

The changes observed in offspring subjected to SR are probably related to increased plasma concentrations of corticosterone. The levels of this hormone were not measured in the current study, although increased secretion of this hormone has been demonstrated in SR conditions 8-10,. Thus, further experiments are needed to confirm this hypothesis.

The present study shows that SR during pregnancy is an additional risk factor for the development of hypertension in progeny. The elevation of blood pressure observed in our experimental model occurred through an increase in the sympathetic tone of the heart and a decrease in cardiac baroreflex control, suggesting that central nervous system changes were involved in fetal programming in this experimental model.

## AUTHOR CONTRIBUTIONS

Gomes GN, Palma BD, Bergamaschi CT and Campos RR conceived and designed the experiments. Raimundo JR performed the experiments. Raimundo JR, Gomes GN, Bergamaschi CT and Campos RR analyzed the data. Gomes GN, Bergamaschi CT, Campos RR and Tufik S contributed with the reagents, materials and analysis tools. Gomes GN, Bergamaschi CT, Campos RR and Raimundo JR wrote the manuscript.

## Figures and Tables

**Table 1:** Body weight (BW) and naso-anal length (NAL) in the studied groups.

	CO		SRO	
Age (months)	BW (g)	NAL (cm)	BW (g)	NAL (cm)
**1**	85.5±2.6	13.3±0.16	85.3±1.43	13.4±0.14
**2**	255.8±7.5	19.1±0.2	254.5±4.36	19.1±0.16
**3**	341.9±8.6	22±0.3	346.2±7.63	21.2±0.2*
**N**	17	17	24	24

Data are reported as the mean±SEM; N = number of animals in each group.

CO = control mother offspring; SRO = sleep-restricted mother offspring.

*p≤0.05 *vs.* CO group (Mann Whitney test).

**Table 2:** Renal function analysis in the studied groups.

	CO	SRO
**GFR (ml/min/Kg)**	7.5±0.2	8.5±0.4*
**RPF (ml/min/Kg)**	21.8±1.6	22.8±1.9
**TA mM/l**	1.3±0.21	1.0±0.33
**EANH4 mM/l**	2.8±0.63	3.5±1.0
**FE Na+ %**	0.75±0.16	0.51±0.15
**FE K+ %**	28.6±2.7	25.6±2.6
**N**	9	8

Data are reported as the mean ± SEM; N = number of animals/group. GFR = glomerular filtration rate, RPF = renal plasma flow, TA = titratable acid, EANH4 = excreted amount of ammonium, FE Na^+^ = fractional excretion of sodium, FE K^+^ = fractional excretion of potassium.

CO = control mother offspring; SRO = sleep-restricted mother offspring.

*p≤0.05 *vs.* CO group (Students T test and Mann Whitney test for RPF).

**Table 3:** Evaluation of sympathetic and parasympathetic tone.

	CO	SRO
**Atenolol Δ (bpm)**	-15±3.27	-40±8.81*
**Atropin Δ (bpm)**	113±14.55	91±14.57
**Hexamethonium Δ (mmHg)**	-38±3.86	-39±3.48
**N**	7	6

Data are reported as the mean±SEM; N = number of animals/group.

CO = control mother offspring; SRO = sleep-restricted mother offspring.

*p≤0.05 *vs.* CO group (Students T test and Mann Whitney test for Hexamethonium Δ).

**Table 4:** Results of spectral analysis.

	CO	SRO
**LF PI (Hz)**	14.08±0.98	25.8±2.1*
**HF PI (Hz)**	85.92±0.98	74.11±2.1*
**LF/HF PI (Hz)**	0.17±0.014	0.38±0.043*
**LF AP (Hz)**	4.48±0.65	7.82±1.09*
**HF AP (Hz)**	2.9±0.65	2.16±0.32
N	12	9

Data are reported as the mean±SEM; N = number of animals/group.

CO = control mother offspring; SRO = sleep-restricted mother offspring.

*p≤0.05 *vs.* CO group (Students T test and Mann Whitney test for HF AP).
